# GLP-1 Receptor Agonists and Kidney Protection

**DOI:** 10.3390/medicina55060233

**Published:** 2019-05-31

**Authors:** Eulalia Valentina Greco, Giuseppina Russo, Annalisa Giandalia, Francesca Viazzi, Roberto Pontremoli, Salvatore De Cosmo

**Affiliations:** 1 Unit of Internal Medicine, Department of Medical Sciences, IRCCS Casa Sollievo della Sofferenza, 71013 San Giovanni Rotondo (FG), Italy; eulaliagreco85@gmail.com; 2 Department of Clinical and Experimental Medicine, University of Messina, 98121 Messina, Italy; giuseppina.russo@unime.it (G.R.); agiandalia@yahoo.it (A.G.); 3 Department of Internal Medicine, University of Genoa and Policlinico San Martino-IST, 16131 Genoa, Italy; francesca.viazzi@unige.it (F.V.); roberto.pontremoli@unige.it (R.P.)

**Keywords:** type 2 diabetes mellitus, diabetic kidney disease, albuminuria, glucagon-like peptide-1 receptor agonist, kidney protection

## Abstract

Type 2 diabetes mellitus (T2DM) is the leading cause of chronic kidney disease (CKD). Diabetic nephropathy (DN) is determined by specific pathological structural and functional alterations of the kidneys in patients with diabetes, and its clinical manifestations are albuminuria and decline of glomerular filtration rate (GFR). Apart from renin–angiotensin–aldosterone system (RAAS) inhibitors, no other drugs are currently available as therapy for diabetic kidney disease (DKD). Glucagon-like peptide-1 receptor (GLP-1R) agonists are a new class of anti-hyperglycemic drugs which have been demonstrated to prevent the onset of macroalbuminuria and reduce the decline of GFR in diabetic patients. These drugs may exert their beneficial actions on the kidneys through blood glucose- and blood pressure (BP)-lowering effects, reduction of insulin levels and weight loss. Clinical benefits of GLP-1R agonists were acknowledged due to data from large randomized phase III clinical trials conducted to assess their cardiovascular(CV) safety. These drugs improved renal biomarkers in placebo-controlled clinical studies, with effects supposed to be independent of the actions on glycemic control. In this review, we will focus on the actions of GLP-1R agonists on glucose metabolism and kidney physiology, and evaluate direct and indirect mechanisms through which these drugs may confer renal protection.

## 1. Introduction

Type 2 diabetes mellitus (T2DM) confers an increased risk of developing macrovascular and microvascular complications, which may result in disability for affected individuals as well as considerable costs for global health-care systems [1]. Moreover, T2DM represents the main cause of chronic kidney disease (CKD) and end-stage renal disease (ESRD), accounting for almost 50% of all patients starting renal replacement therapy (RRT) worldwide [2]. At least half of patients with T2DM will develop diabetic kidney disease (DKD), characterized clinically by a persistent reduction in estimated glomerular filtration rate (eGFR) and/or increased urinary excretion of albumin (micro- or macro-albuminuria). DKD is the single strongest predictor of morbidity and premature mortality in patients with diabetes [3]. Even mild increases in albuminuria or GFR decline are associated with a considerably increased risk of cardiovascular diseases (CVDs) and CV death, with consequent increased health-care costs [4]. Therefore, kidney protection is a critical target in T2DM. 

Diabetic nephropathy (DN) refers to specific pathologic structural and functional alterations of kidneys in patients with diabetes [5]. Glomerular alterations consist of glomerular basement membrane thickening, mesangial expansion, increased matrix secretion, and interstitial fibrosis, produced by activation of myofibroblasts and inflammatory cells, with deposition of atypical collagen and loss of capillary architecture. There are also vascular lesions, including arterial hyalinosis [6]. Hyperglycemia plays a critical role in DKD initiation; it provokes cellular alterations through altered ratios of glucose metabolites, fatty acids, and amino acids, modifications of mitochondrial respiratory chain function, and uncoupling of the respiratory chain proteins [7]. The clinical manifestations are proteinuria, hypertension, and progressive decrease of renal function [6].

Since the development of renin–angiotensin–aldosterone system (RAAS) inhibitors, no new drugs have received regulatory approval for DKD therapy so far [8]. Consequently, new therapeutic agents for prevention of development and progression of DKD represent an urgent medical need. In the last decade, three new classes of anti-hyperglycemic drugs with putative glucose-independent effects have been successfully introduced for the management of T2DM: glucagon-like peptide-1 receptor (GLP-1R) agonists, dipeptidyl peptidase-4 (DPP-4) inhibitors, and sodium–glucose cotransporter 2 (SGLT2) inhibitors [9]. Recently, several clinical trials have reported that GLP-1R agonists are able to prevent the onset of macroalbuminuria and reduce the decline in eGFR in patients affected by T2DM. While SGLT2 inhibitors might have beneficial effects on the kidneys because they reduce glomerular hyperfiltration and restore tubule-glomerular feedback [10], the possible protective mechanisms of GLP-1R agonists on kidneys are less acknowledged.

In our review, we discuss the effects of GLP-1 and GLP-1R agonists on glucose metabolism and kidney physiology and evaluate direct and indirect pathways by which these drugs may confer renal protection.

## 2. Incretin Axis and Glucagon-Like Peptide-1 (GLP-1)

Incretins are peptides produced by the cells of the small intestine in response to the ingestion of various nutrients. Two incretin hormones have been identified: the glucose-dependent insulinotropic polypeptide (GIP) is produced by entero-endocrine K cells, whereas GLP-1 is mainly secreted from L cells located throughout the intestine, more abundantly towards the distal ileum and colon. K cells and L cells are directly stimulated by luminal glucose through the sodium–glucose cotransporter 1 (SGLT1) inhibitor and release incretins in response to other nutrients. Incretins participate in the regulation of carbohydrate metabolism through increasing the production of insulin in a glucose-dependent manner and suppressing endogenous glucagon release by pancreatic α-cells. Moreover, incretins improve glucose sensitivity in pancreatic β-cells, stimulate β-cell proliferation, and reduce their apoptosis [11].

GLP-1 seems also to play a role in the central regulation of feeding through increasing satiety signals and reducing appetite, resulting in a decrease in food intake and weight loss. Furthermore, GLP-1 exerts effects on the gastrointestinal tract by slowing the gastric emptying rate and small intestinal peristalsis, and by reducing pancreatic exocrine secretion of bile acids, digestive enzymes, and bicarbonate.

Circulating GLP-1 has a very short half-life (less than 2 min) and is rapidly inactivated, mostly by the ubiquitous proteolytic enzyme DPP-4 and to a lesser degree by other endopeptidases and aminopeptidases [12]. GLP-1 exerts its glucose-lowering actions through activating its specific receptor GLP-1R. There are two classes of incretin-based therapies for the management of hyperglycemia in T2DM: GLP-1R agonists and DPP-4 inhibitors [9]. We will focus on the actions of GLP-1R agonists. Several GLP-1R agonist formulations have been introduced for management of hyperglycemia in T2DM. Short-acting GLP-1R agonists (exenatide and lixisenatide) mainly lower post-prandial glucagon and significantly delay gastric emptying, subsequently reducing postprandial glucose levels and excursions of insulin levels [13]. Long-acting GLP-1R agonists (such as albiglutide, dulaglutide, exenatide long-acting release, liraglutide, and semaglutide) stimulate insulin secretion and reduce glucagon levels, thus decreasing preeminently fasting plasma glucose. [9,11]. In most studies, GLP-1R agonists reduced hemoglobin A1c (HbA1c) to 0.8–1.5% (8–15 mmol/mol) from baseline values of 7.5–8.5% (58–68 mmol/mol) [14]. They do not affect gastric motility after prolonged administration due to tachyphylaxis [11].

## 3. Effects GLP-1R Agonists on Kidney Protection in Type 2 Diabetes Mellitus (T2DM): Putative Direct and Indirect Actions of GLP-1R Agonists on the Kidneys

GLP-1R agonists may exert beneficial effects on traditional risk factors for CKD, for example through lowering glucose and blood pressure (BP), decreasing insulin levels, and causing weight loss [15].

GLP-1R has been localized at various sites in kidneys. Studies conducted in animal models reported the presence of GLP-1R mRNA in the glomerulus and the initial part of proximal convoluted tubes, with no mRNA expression in other sites of the nephron [16]. Other research detected mRNA in glomeruli, but not in tubules [17]. In humans, GLP-1R has been identified in the kidney localized in proximal tubular cells and preglomerular vascular smooth muscle cells [18].

Emerging evidence suggests the potential protective actions on kidneys of GLP-1R agonists, independently of their glucose lowering effects, some of which may play a role in inhibition of development and progression of DKD [19]. Therefore, the potential for GLP-1R agonists to positively affect renal risk factors in T2DM [15] might translate to better clinical outcomes beyond glycemic control.

GLP-1 has been demonstrated to induce natriuresis and diuresis, likely involving the inhibition of the sodium–hydrogen exchanger 3 (NHE3) localized at the brush border of the renal proximal tubular cells [20]. This effect may partially explain the BP-lowering effects of GLP-1R agonists. The acute administration of GLP-1R agonists likely increases the effective renal plasma flow and the GFR, at least in healthy individuals [21], determined by a temporal increase in BP, which in turn results from an increase in heart rate and cardiac output. Chronic administration of GLP-1R agonists reduces BP, increases natriuresis, and influences renal risk factors supporting the maintenance of renal function [22].

Several molecules playing a role in glucose metabolism including insulin, ATP, and glucose itself regulate NHE3 and SGLTs in the kidney, thus suggesting some indirect natriuretic actions of GLP-1 [23].

The activation of the intrarenal RAAS is a well-known pathogenic mediator of DN and there is clear evidence of the renoprotective efficacy of RAAS blockade in DKD. In experimental studies, GLP-1 and GLP-1R agonists have been demonstrated to reduce markers of renal RAAS activation, including angiotensin II levels and its deleterious effects in the glomerulus, which may represent other potential renoprotective mechanisms in DKD [24]. However, no conclusive data are available to confirm effects of acute or prolonged GLP-1R agonist treatment on circulating RAAS components. 

**Figure 1:**
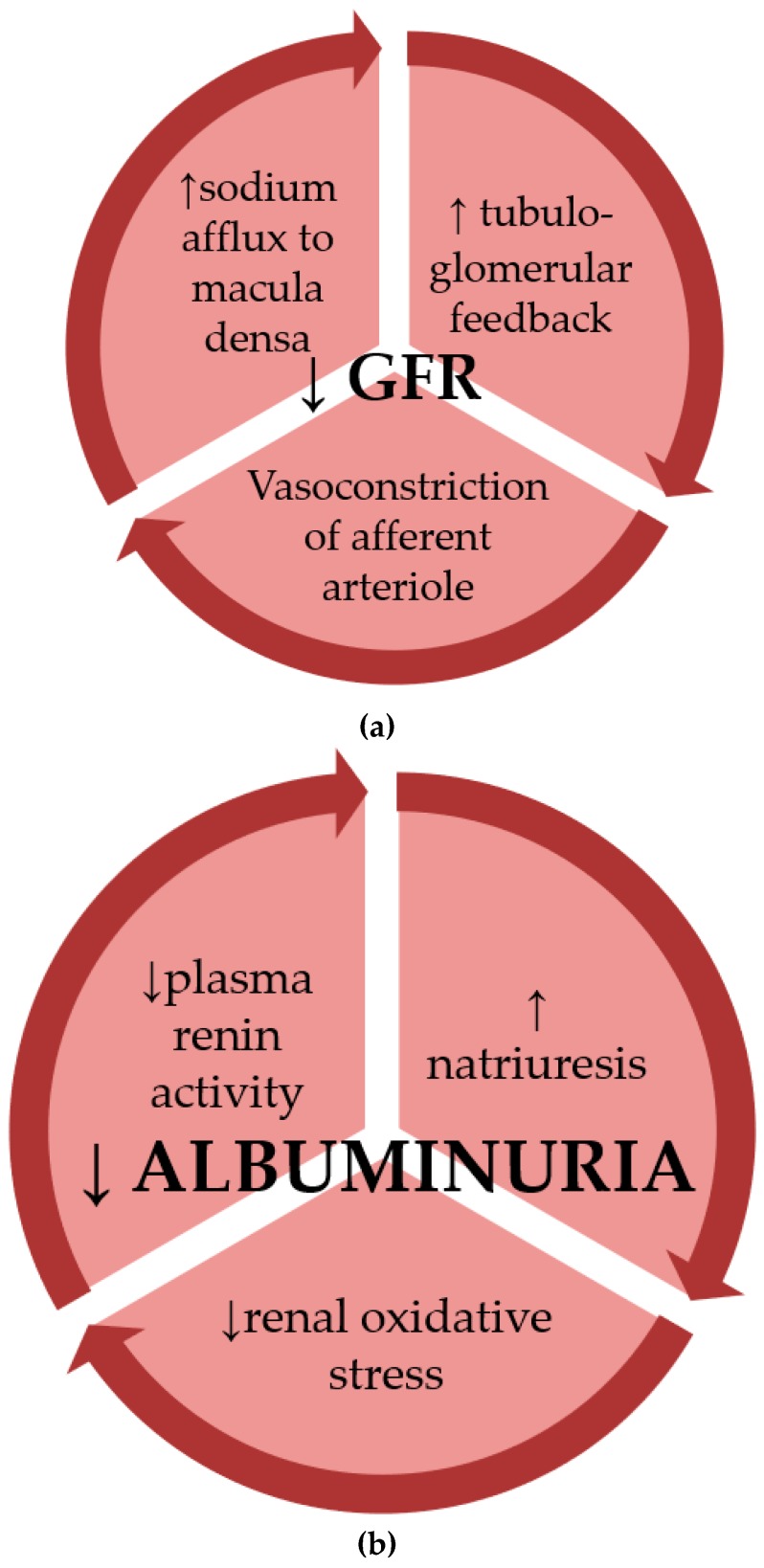
(**a**) Chronic administration of glucagon-like protein-1 receptor (GLP-1R) agonists has been shown to affect renal hemodynamics through decreasing the estimated glomerular filtration rate (eGFR). (**b**) The antialbuminuric actions of GLP-1R agonists on kidneys involve effects on multiple mechanisms of diabetic nephropathy (DN).

GLP-1 induces glomerular hyperfiltration under physiological conditions; the mechanisms involved might be the increase of the filtered electrolyte load after food ingestion, or the reduction of afferent arteriolar resistance. However, GLP-1R agonists might slightly improve renal hemodynamic function in patients with T2DM by inhibition of pathways of glomerular hyperfiltration (Figure 1a). 

The natriuretic effect of GLP-1R agonists along with the effects of these drugs on the conventional risk factors for DN (such as hyperglycemia, hypertension and obesity) may represent the most relevant mechanisms underlying their antialbuminuric effect (Figure 1b).

GLP-1R agonist therapy determines a small reduction in the levels of low-density lipoprotein (LDL) cholesterol, total cholesterol and triglycerides, but does not improve high-density lipoprotein (HDL) cholesterol [25]. The mechanisms by which GLP-1R agonists improve dyslipidemia are only partially known. GLP-1R agonists have demonstrated to reduce production and secretion of intestinal chylomicrons thus contributing to reduce absorption and circulating levels of triglycerides [26]. Finally, studies on rodent models have shown that GLP-1R agonists seem to help clearance of lipids from the circulation by activating brown adipose tissue, which produces heat by burning triglycerides [27].

Although diabetes is not considered an immune disease, there is increasing evidence supporting a role for inflammation in diabetes. Inflammatory cells, cytokines, and profibrotic growth factors have all been involved in the pathogenesis of DN via increased vascular inflammation and fibrosis. Recent evidence demonstrates that GLP-1 modulates inflammation at multiple sites, including the kidneys and blood vessels [28]. The cyclic adenosine monophosphate–protein kinase A (cAMP–PKA) pathway activation is able to reduce ROS production in the diabetic kidney. Activation of GLP-1R leads to stimulation of cAMP–PKA pathways, having antioxidative effects; thus, it is likely that GLP-1 protects the kidney from oxidative injury [29].

In high-fat diet rats (HFD), liraglutide restored kidney mitochondrial function counteracting the decline of biogenesis of genes encoding for proteins of mitochondrial respiration and increasing the uncoupling protein 2 levels [30]. Authors concluded that liraglutide could directly prevent lipid deposition in the kidney of HFD rats by coordinating lipogenic and lipolytic factors. Therefore, GLP-1 has demonstrated a role in renal protection trough modulation of lipid and energy metabolism, and it is supposed that GLP-1R agonists may be a promising therapy in obesity-associated CKD.

It has recently been proposed that DKD may be considered, at least partially, a renal manifestation of dyslipidemia and atherosclerosis [31]. GLP-1R agonists have demonstrated to have anti-atherogenic effects, both indirectly by modulating glucose and lipid metabolism, weight, and BP, and probably through direct anti-inflammatory and anti-ischemic actions [32].

Fujita et al. [33] detected GLP-1R expression in glomerular capillary and vascular walls within a mouse kidney model of DN. To explore whether the presence or absence of GLP-1R signaling had a crucial role in the development and progression of DN, the authors disrupted the *Glp1r* gene in the DN-resistant mouse model and investigated its renal phenotype. The loss of the GLP-1R resulted in elevation of glomerular superoxide and renal oxidative stress. These renal modifications consequently to GLP-1R absence contributed to the development of DN. In this study the GLP-1R agonist liraglutide was shown to ameliorate to ameliorate the oxidative stress through increasing cAMP levels and PKA activity and reducing NAD(P)H oxidase activity in nephropathy-prone mice kidneys. Additionally, liraglutide inhibited the progression of DKD by reducing mesangial expansion and increasing glomerular nitric oxide levels, improving glomerular hyperfiltration. Remarkably, renal improvement has been obtained without major changes in insulin secretion or glucose tolerance, thus supporting direct renal effects. Together, these findings support the hypothesis that GLP-1R signaling may directly exert antioxidant and protective effects on the diabetic kidney.

GLP-1R-induced cAMP activation might also result in reduced expression of the receptor of advanced glycation end products (AGEs). In rodent models of diabetes, GLP-1 has been shown to interfere with the signaling and expression of the receptor for AGEs, resulting in antioxidative effects [34].

Some studies have described that treatment with GLP-1R agonists is able to modulate the microbiome in mice. However, the exact mechanism is unclear and obviously may be a result of modifications in food intake and diet following start of GLP-1R agonist therapies. Nonetheless, there are some data linking the composition of the gut microbiome with kidney disease [35].

The potential renoprotective effects of GLP-1R agonists are summarized in Table 1.

All these data for the basis for future clinical studies investigating whether GLP-1R agonists will improve renal outcomes in the setting of DKD.

## 4. Evidence from Outcomes of Cardiovascular Safety Studies with GLP-1R Agonists

Clinical benefits of these drugs on DKD were recognized within data resulting from large randomized phase III clinical trials conducted to assess their CV safety with the aim of satisfying a regulatory requirement for drug approval imposed by the 2008 United States Food and Drug Administration industry guidance. GLP-1R agonists improved renal biomarkers in placebo-controlled clinical studies, an effect supposed to be independent of the actions of these drugs on glycemic control.

In the Liraglutide Effect and Action in Diabetes: Evaluation of Cardiovascular Outcome Results (LEADER) trial, 9,340 T2DM patients with CVD or at high CV risk were randomly assigned to liraglutide or placebo. There was a significant difference in HbA1c (−0.4%) and body weight (−2.3 kg) between the two study arms. Liraglutide reduced new or worsening nephropathy by 22% in the LEADER trial after 3.8 years, (hazard ratio (HR) = 0.74 (0.60–0.91)) [36]. These results are consistent with previous small studies supporting a reduction in albuminuria with liraglutide in patients with T2DM [37,38]. Moreover, liraglutide slightly slowed the decline in eGFR over time compared to placebo, with a baseline to month 36 ratio of 0.89 with liraglutide and 0.88 with placebo (difference of 1.015, *p* = 0.013). Subgroup analyses revealed that this decline occurred mainly in patients with macroalbuminuria or an eGFR 30–59 mL/min/1.73 m^2^ at baseline. In contrast, the doubling of serum creatinine concentration to an eGFR ≤45 mL/min/1.73 m² was unaffected (HR = 0.88 (0.66–1.18). Indeed, no changes have been identified in “hard” renal outcomes, although the incidence of ESRD or renal death were small and the study was underpowered to detect a clear difference in these parameters. Notably, patients with an eGFR < 60 mL/min/1.73 m^2^ appeared to have a significantly greater CV benefit from treatment with liraglutide (HR = 0.69 (0.57–0.85)) than those with an eGFR >60mL/min/1.73 m^2^ (HR = 0.94 (0.83–1.07)). This result was partly driven by the high event rate in patients with CKD, which was almost twice than in patients with normal renal function [39].

The data from the Efficacy and Safety of Liraglutide Versus Placebo as Add-on to Glucose-Lowering Therapy in Patients With Type 2 Diabetes and Moderate Renal Impairment (LIRA RENAL) trial, which investigated the effects of liraglutide in patients with T2DM with moderate renal impairment, showed that liraglutide did not affect eGFR after 26 weeks [40].

In the Trial to Evaluate Cardiovascular and Other Long-term Outcomes with Semaglutide in Subjects with Type 2 Diabetes (SUSTAIN-6), 3,297 patients with T2DM and CVD or with CV risk factors were randomly allocated to receive semaglutide (at the dose of 0.5 or 1 mg once weekly) or placebo. This strategy resulted in a significant difference in glycemic control (−0.7% and −1.0% of HbA1c) and body weight (−2.9 kg and −4.3 kg) between the two study arms [41]. After a median follow-up of two years, new or worsening nephropathy occurred less often in patients treated with semaglutide (HR = 0.64 (0.46–0.88), *p* = 0.005). As was seen in LEADER, this renal outcome was also driven by a reduction in new onset macroalbuminuria (semaglutide vs. placebo; 2.5% vs. 4.9%, respectively). Doubling of serum creatinine concentration to an eGFR ≤45 mL/min/1.73 m², ESRD, or renal death were unaffected, although again the event rate was too low (<1%) to adequately explore these outcomes. It was found that 104 weeks of semaglutide treatment reduced this composite outcome by 36%. 

In particular, the renal benefit in LEADER was primarily driven by a 26% reduction in macroalbuminuria, while in SUSTAIN-6, an even larger 46% reduction in macroalbuminuria seems to be exclusively responsible for the better renal outcome of semaglutide. Both LEADER and SUSTAIN-6 had a prespecified composite renal outcome of new or worsening nephropathy consisting of new onset of persistent macroalbuminuria, persistent doubling of serum creatinine level and eGFR ≤45 mL/min/1.73 m^2^, need for continuous RRT (in absence of an acute reversible cause), and renal death.

In the trial Evaluation of Cardiovascular Outcomes in Patients With Type 2 Diabetes After Acute Coronary Syndrome During Treatment With Lixisenatide (ELIXA), 6068 patients with T2DM and acute coronary syndrome were randomized to receive lixisenatide or placebo. Differently from the treatment with GLP-1R agonists in SUSTAIN-6 and LEADER, lixisenatide determined a more modest difference in glycemic control (−0.27% of HbA1c) and body weight (−0.7 kg) compared to placebo. In ELIXA, even if the pre-specified analysis of the percentage change in the urinary albumin-to-creatinine ratio (UACR) from baseline to 108 weeks showed a modest difference in favor of lixisenatide over placebo (24% vs. 34%, *p* = 0.004), the median values at baseline (ratio, 10 in each study treatment group) and follow-up (ratio, 12 in the lixisenatide group and 13 in the placebo group) were clinically almost similar [42]. Post-hoc adjustments for slight differences in HbA1c levels (~0.3%) during the first 3 months of the trial attenuated the lixisenatide-induced renal benefit (*p* = 0.07), suggesting some glucose-dependency.

However, in a recently published exploratory analysis of ELIXA [43], Muskiet et al. examined the effect of lixisenatide on renal outcomes. Lixisenatide was associated with a reduced risk of new-onset macroalbuminuria compared with placebo when adjusted for baseline HbA1c (HR = 0.808 (95% confidence interval (CI) 0.660–0.991; *p* = 0.0404)) and on-trial HbA1c (HR = 0.815 (0.665–0.999; *p* = 0.0491)). At week 108, the largest eGFR decline from baseline was observed in the macroalbuminuric group, but no significant differences were observed between the two treatment groups. Moreover, no significant differences in eGFR decline were detected between treatment groups in any UACR subgroup. Doubling of serum creatinine occurred in 35 (1%) of 3032 patients in the placebo group and 41 (1%) of 3031 patients in the lixisenatide group (HR 1.163, (95% CI 0.741–1.825); *p* = 0.5127)). As described in the ELIXA trial, the proportion of patients with renal adverse events was low (48 (1.6%) of 3,032 patients in the placebo group vs. 48 (1.6%) of 3,031 patients in the lixisenatide group) and did not significantly differ between treatment groups. The beneficial effect of lixisenatide on UACR in ELIXA was observed after adjustment for on-trial HbA1c and other traditional metabolic and hemodynamic risk factors. This explorative analysis suggests that short-acting GLP-1R agonists might have similar renal effects as long-acting GLP-1R agonists, which have been reported in previous CV outcome trials in this target population. However, it is noteworthy that the larger effect on SBP observed in the SUSTAIN-6 trial (−1.3 mmHg and −2.4 mmHg, respectively) compared to ELIXA (−0.8 mmHg) may have also contributed to the larger reduction of macroalbuminuria detected in the SUSTAIN-6.

In the trial Exenatide Study of Cardiovascular Event Lowering (EXSCEL) 14,752 patients affected by T2DM with (73.1%) or without previous CVD, were randomized to receive subcutaneous injections of extended-release exenatide at a dose of 2 mg or placebo once weekly and followed for a median of 3.2 years. The trial demonstrated the CV safety of exenatide once-weekly [44]. Bethel et al. investigated in an adjusted analysis the renal outcomes of EXSCEL trial. They reported that a composite of 40% eGFR decline, renal replacement, renal death or new macroalbuminuria was significantly reduced through the addition of exenatide in people with T2DM (HR 0.85 (95% CI 0.73–0.98, *p* = 0.027)) [45].

It is also plausible that the major weight loss achieved in phase III clinical trials with GLP-1RAs described above also contributed to their effects on albuminuria, and the comparatively superior effect of semaglutide on both weight loss and new onset nephropathy. However, it must be emphasized that the rate of these endpoints in these trials was very small.

A Study Comparing Dulaglutide With Insulin Glargine on Glycemic Control in Participants With Type 2 Diabetes (T2D) and Moderate or Severe Chronic Kidney Disease (CKD) (AWARD-7), was a multicenter and open-label trial conducted on 577 patients with T2DM and moderate to-severe CKD (stages 3-4). Participants were randomized to receive (1:1:1) once-weekly injectable dulaglutide 1.5 mg, once-weekly dulaglutide 0.75 mg, or daily insulin glargine as basal therapy, all in combination with insulin lispro, for 52 weeks. The primary outcome was HbA1c at 26 weeks, with a 0.4% non-inferiority margin. Secondary outcomes included eGFR and UACR values. At 52 weeks, eGFR levels resulted higher with dulaglutide 1.5 mg (least squares mean (LSM) 34.0 mL/min/1.73 m^2^ (standard error (SE) 0.7); *p* = 0.005 vs. insulin glargine) and dulaglutide 0.75 mg (33.8 mL/min/1.73 m^2^ (0.7); *p* = 0.009 vs. insulin glargine) than with insulin glargine (31.3 mL/min/1.73 m^2^ (0.7)). At 52 weeks, the effects of dulaglutide 1.5 mg and 0.75 mg on UACR reduction did not significantly differ from that of insulin glargine (LSM −22.5% (95% CI −35.1 to −7.5) with dulaglutide 1.5 mg; −20.1% (−33.1 to −4.6) with dulaglutide 0.75 mg; −13.0% (−27.1–3.9) with insulin glargine). ESRD occurred in 38 participants: 8 (4%) of 192 with dulaglutide 1.5 mg, 14 (7%) of 190 with dulaglutide 0.75 mg, and 16 (8%) of 194 with insulin glargine [46]. The results of trial AWARD-7 showed that once-weekly dulaglutide produced clinically meaningful improvements in glycemic control in T2DM patients with moderate-to-severe CKD, with efficacy similar to that of daily insulin glargine as basal therapy in terms of change in HbA1c. The analysis of secondary endpoints suggested that dulaglutide attenuates eGFR decline compared with insulin glargine in patients with T2DM and moderate-to-severe CKD after 52 weeks. This was the first clinical trial in patients with T2DM and moderate-to-severe CKD that has shown clear effects of a GLP-1R agonist on eGFR.

The phase III cardiovascular outcomes trial for dulaglutide Researching Cardiovascular Events with a Weekly Incretin in Diabetes (REWIND) has just concluded and was associated with significantly reduced major adverse CV events; its results have not been published. 

The renal endpoints and the exploratory kidney outcomes of the above cited trials are summarized in Table 2.

The recently published Albiglutide and cardiovascular outcomes in patients with type 2 diabetes and cardiovascular disease (Harmony Outcomes) is a double-blind, randomized, placebo-controlled trial conducted on 9,463 T2DM subjects with CVD, with a median follow-up of 1.6 years. Participants were randomized (at a 1:1 ratio) to receive a subcutaneous injection of albiglutide (30–50 mg, based on glycemic response and tolerability) or of a matched volume of placebo once-weekly, in addition to their standard care. Albiglutide was superior to placebo with respect to major adverse CV events, reducing the risk of the primary composite outcome (death from CV causes, non-fatal myocardial infarction, or non-fatal stroke) by 22% compared to placebo [47]. There are no available subgroup analyses on renal outcomes so far.

The effect of GLP-1R agonists on eGFR decline and hard renal endpoints (ESRD or eGFR <15 mL/min/1.73 m^2^) still remains unknown, and should be explored in dedicated renal outcome studies with longer follow-up times in patients with T2DM and more advanced CKD at baseline. 

## 5. Conclusions and Future Perspectives

As insulin plays a direct role in the development and progression of DKD, GLP-1R agonists may have beneficial effects for various organs including the kidneys, reducing insulin levels and improving insulin sensitivity. The effects on reducing BP may contribute to the anti-albuminuric effects of GLP-1R agonists in patients with T2DM. 

Protecting the kidneys is a critical target in T2DM. The effect of GLP-1R agonists on eGFR decline and hard renal endpoints (e.g., ESRD or eGFR < 15 mL/min/1.73 m^2^) remains equivocal, and should be investigated in dedicated renal outcome studies with longer follow-up periods in patients with T2DM and more advanced CKD at baseline.

There is now satisfactory evidence to demonstrate that treatment with GLP-1R agonists is able to reduce albuminuria. As albuminuria is a strong predictor of renal outcomes, its decline may represent a valid indicator of a reduction in renal risk [48]. However, clear evidence for effects on hard renal outcomes is lacking, mainly because very few patients with advanced renal disease receive GLP-1R agonists because of poor tolerability in this setting. As both experimental and clinical data now support the possibility of renoprotective effects arising from treatment with GLP-1R agonists, further research is required to extend current knowledge of the underlying mechanisms.

## Figures and Tables

**Table 1:** Putative renoprotective actions and effects of GLP-1R agonists on kidneys.

Direct Effects	Indirect Effects
Proximal tubular natriuresis stimulation	Improved glycemic control
Modulation of cAMP/PKA signaling	Improved blood pressure control
Inhibition of renin angiotensin system	Weight loss
↓ Renal hypoxia	↑ Insulin sensitivity
↓ Glomerular atherosclerosis?	↓ Postprandial glucagon
Renal endothelial dependent vasodilation	↓ Intestinal lipid uptake
↑ Tubuloglomerular feedback (through ↓ NHE3 activity)	↑ Brown adipose tissue activation
↑ ANP?	Effects on microbioma?

Abbreviations— GLP-1R: glucagon like peptide-1 receptor; cAMP: cyclic adenosine monophosphate; PKA:protein kinase A; NHE3:sodium–hydrogen exchanger 3; ANP:atrial natriuretic peptide.

**Table 2:** Renal outcomes in clinical trials with GLP-1R agonists in patients with T2DM.

Name of the Study	Drug and Intervention	Study Population	Renal Endpoints	Exploratory Kidney Outcomes (Change from Baseline Placebo/Intervention or HR 95% CI)	Results
ELIXA	10–20 μg of Lixisenatide versus placebo	6068 patients with a myocardial infarction or hospitalization for unstable angina within the previous 180 days with a median follow-up of 108 weeks	Change in UACR (%) from baseline to 108 weeks	24% vs. 34%, *p* = 0.004	Lixisenatide reduces progression of UACR in macroalbuminuric patients, with a lower risk of new-onset macroalbuminuria
			% Change in UACR in		
			normoalbuminuria	−1.69% (11.69–8.30; *p* = 0.7398)	
			microalbuminuria	−21.10% (42.25–0.04; *p* = 0.0502)	
			macroalbuminuria	−39.18% (68.53–9.84; *p* = 0.007)	
			Risk of new-onset macroalbuminuria	0.808 (0.660–0.991; *p* = 0.0404)	
LEADER	Liraglutide 1.8 mg (or the maximum tolerated dose) versus placebo	9340 patients high CV risk with a median follow-up of 3.84 years	Composite end point	0.78 (0.67–0.92, *p* = 0.003)	Liraglutide determined a lower risk of the composite renal outcome than placebo, mainly owing to a lower rate of new-onset persistent macroalbuminuria.
			New-onset persistent albuminuria	0.74 (0.60–0.91, *p* = 0.004)	
			Persistent doubling of sCr and eGFR <45 mL/min/1.73 m^2^	0.89 (0.67–1.19, *p* = 0.43)	
			Need for continuous RRT	0.87 (0.61–1.24, *p* = 0.44)	
			Death due to renal disease	1.59 (0.52–4.87, *p* = 0.41)	
SUSTAIN-6	Semaglutide 0.5 mg vs. 1.0 mg vs. placebo	3297 patients, 83% had established CVD, CKD, or both. The median follow-up was 108 weeks	New or worsening nephropathy	0.64 (0.46–0.88, *p* = 0.005)	The reduction in macroalbuminuria seems exclusively responsible for the favorable renal outcome of semaglutide
			New onset of persistent macroalbuminuria	0.54 (0.37–0.77, *p* = 0.001)	
			Persistent doubling of the sCr and a eGFR <45 mL/min/ 1.73 m^2^	1.28 (0.64–2.58, *p* = 0.48)	
			Need for continuous RRT	0.91 (0.40–2.07, *p* = 0.83)	
AWARD-7	Dulaglutide 0.75 and 1.5 mg vs. insulin glargine	577 patients with moderate-to-severe CKD with a follow-up of 52 weeks	eGFR and UACR change from baseline	eGFR with dulaglutide 1.5 mg 34.0 mL/min/1.73 m² and (SE 0.7); *p* = 0.005 vs. insulin glargine;	Dulaglutide produced glycemic control similarly to insulin glargine, with reduced decline in eGFR. Dulaglutide appears to be safe to achieve glycemic control in patients with moderate-to-severe CKD.
				dulaglutide 0.75 mg (33.8 mL/min/1.73 m² (0.7); *p* = 0.009 vs. insulin glargine);	
				insulin glargine (31.3 mL/min/1.73 m² (0.7)).	
				UACR reduction with dulaglutide 1.5 mg −22.5% [95% CI −35.1 to −7.5], −20.1% (−33.1 to −4.6] with dulaglutide 0.75 mg; −13.0% (−27.1–3.9) with insulin glargine	
EXSCEL	Extended-release exenatide 2 mg or placebo once weekly	14,752 patients (of whom 73.1% had previous CVD) followed for a median of 3.2 years	Renal composite 2 (40% eGFR decline, renal replacement, renal death or new macroalbuminuria)	0.85 (0.73–0.98, *p* = 0.027)	A composite of 40% eGFR decline, renal replacement, renal death or new macroalbuminuria was significantly reduced in an adjusted analysis by the addition of exenatide people with T2DM. Other renal outcomes were numerically but not statistically improved with exenatide.
LIRA-RENAL	Liraglutide 1.8 mg versus placebo	279 patients with moderate renal impairment (eGFR 30–59 mL/min/1.73 m^2^)	Change in HbA1c from baseline to week 26	−1.05% vs. −0.38%, respectively	Liraglutide did not affect renal function and proved better glycemic control, with no increase in hypoglycemia, but with higher withdrawals due to gastrointestinal adverse events than placebo in people with T2DM and moderate renal impairment.

Clinical trials for glycemic control in moderate-to-severe CKD: AWARD-7, LIRA-RENAL. Clinical trials for CV safety: LEADER, ELIXA, SUSTAIN-6, and EXSCEL. Abbreviations—GLP-1R: glucagon like peptide-1 receptor; T2DM: type 2 diabetes mellitus; HR: hazard ratio; CI: confidence interval; UACR: urinary albumin-to-creatinine ratio (albumin measured in mg/g); CV: cardiovascular; sCr; serum creatinine; eGFR: estimated glomerular filtration rate in mL/min/1.73 m^2^; CKD: chronic kidney disease; CVD: CV disease; CrCl: creatinine clearance; RRT: renal replacement therapy; SE: standard error; HbA1c: hemoglobin A1c.
